# Prediction of the number of asthma patients using environmental factors based on deep learning algorithms

**DOI:** 10.1186/s12931-023-02616-x

**Published:** 2023-12-01

**Authors:** Hyemin Hwang, Jae-Hyuk Jang, Eunyoung Lee, Hae-Sim Park, Jae Young Lee

**Affiliations:** 1 Environmental Engineering Department, Ajou University, Suwon, 16499 Korea; 2 Department of Allergy and Clinical Immunology, Ajou University School of Medicine, Suwon, 16499 Korea; 3 Department of Neurology, McGovern Medical School, The University of Texas Health Science Center at Houston, Houston, TX 77030 USA; 4 Environmental and Safety Engineering Department, Ajou University, 206, World Cup-ro, Yeongtong-gu, Suwon, 16499 Korea

**Keywords:** Recurrent neural network, Long short-term memory, Gated recurrent unit, Air pollution, Asthma, Influenza

## Abstract

**Background:**

Air pollution, weather, pollen, and influenza are typical aggravating factors for asthma. Previous studies have identified risk factors using regression-based and ensemble models. However, studies that consider complex relationships and interactions among these factors have yet to be conducted. Although deep learning algorithms can address this problem, further research on modeling and interpreting the results is warranted.

**Methods:**

In this study, from 2015 to 2019, information about air pollutants, weather conditions, pollen, and influenza were utilized to predict the number of emergency room patients and outpatients with asthma using recurrent neural network, long short-term memory (LSTM), and gated recurrent unit models. The relative importance of the environmental factors in asthma exacerbation was quantified through a feature importance analysis.

**Results:**

We found that LSTM was the best algorithm for modeling patients with asthma. Our results demonstrated that influenza, temperature, PM_10_, NO_2,_ CO, and pollen had a significant impact on asthma exacerbation. In addition, the week of the year and the number of holidays per week were an important factor to model the seasonality of the number of asthma patients and the effect of holiday clinic closures, respectively.

**Conclusion:**

LSTM is an excellent algorithm for modeling complex epidemiological relationships, encompassing nonlinearity, lagged responses, and interactions. Our study findings can guide policymakers in their efforts to understand the environmental factors of asthma exacerbation.

**Supplementary Information:**

The online version contains supplementary material available at 10.1186/s12931-023-02616-x.

## Background

Asthma is one of the most prevalent respiratory diseases that have a significant public health burden. According to the Global Asthma Report 2022, 262 million people were affected by asthma, and 461 thousand people died from asthma worldwide in 2019 [1]. In addition, asthma is a chronic disease that seriously reduces patients’ quality of life but has no definitive cure [2].

Due to the severity of asthma, many previous studies have attempted to understand the risk factors that exacerbate asthma, and various environmental factors such as air pollution, tobacco smoke, weather, allergens such as pollen, and pathogens such as influenza viruses have been identified as culprits for asthma exacerbation [3]. These studies mostly used regression-based statistical models such as the generalized linear model (GLM) [4], generalized additive model (GAM) [5], and distributed lag nonlinear model (DLNM) [6] and ensemble-based machine learning models such as the random forest (RF) [7] and gradient boosting machine (GBM) [8]. Cassino et al. analyzed tobacco use and O_3_-associated emergency room visits for asthma in New York City based on a Poisson regression model [9]. Lee et al. studied the effects of air pollutants, pollen, weather conditions, and viruses on the number of emergency room patients with asthma in Seoul, South Korea, using DLNM [10]. Chen et al. studied the lagged nonlinear relationship between temperature and adult asthma hospitalizations in Beijing using the DLNM [2]. Sun et al. studied the association between pollen (trees, weeds, and grasses) and asthma in North Carolina using DLNM [11]. Jeddi et al. compared machine learning models for pediatric asthma diagnosis by considering environmental factors such as mites, cold air, strong odors, and mold [12]. Although previous studies have succeeded in identifying risk factors and modeling the risk of asthma using conventional statistical and machine learning algorithms, our understanding and modeling accuracy remain insufficient because of the complexities associated with nonlinearity, lagged relationships, interactions between factors, multicollinearity, and various confounders.

To model the relationship with higher accuracy, researchers have started to utilize state-of-the-art deep learning algorithms such as recurrent neural networks (RNNs) [13], long short-term memory (LSTM) [14], and gated recurrent units (GRUs) [15]. Woo et al. predicted the peak expiratory flow rate in children with asthma using real-time indoor air pollution data using RNN, GRU, and deep neural network [16]. Kim et al. studied the association between indoor particulate matter (PM) and asthma attacks in children in South Korea using the LSTM [17]. Chang and Ku used LSTM to predict the daily number of patients with asthma affected by weather and air pollution in Seoul, South Korea [18]. As research based on deep learning algorithms in the field of public health is still in its early stages, more research on modeling methodologies and epidemiological results from the models is necessary.

This study examined the association between the number of patients with asthma and 18 environmental factors in South Korea between 2015 and 2019 using the RNN, LSTM, and GRU algorithms. Eighteen environmental factors were categorized into air pollution, weather, pollen, and influenza. The accuracy of the model developed in this study was compared with that of conventional algorithms (GLM, GAM, RF, and GBM), and permutation feature importance analysis was performed to identify the critical factors in asthma exacerbation and understand the interaction between various factors.

## Methods

### Data collection

Weekly counts of patients with asthma in South Korea from 2015 to 2019 were collected by the Health Insurance Review and Assessment Service. Patients with asthma were defined as those aged 17 years or older who visited a healthcare facility and were diagnosed with asthma (ICD-10 codes J45, J46, J820, and J828). The number of outpatients and emergency room (ER) patients with asthma was determined separately. Environmental data were collected from South Korea from 2015 to 2019. Daily air pollutant concentrations of CO, NO_2_, O_3_, PM_10_, PM_2.5_, and SO_2_ were collected from 556 nationwide measurement stations by the Korea Environment Corporation. Daily meteorological data on mean temperature, minimum temperature, maximum temperature, diurnal temperature range, humidity, precipitation, solar radiation, and wind speed were collected from 100 measurement stations in South Korea by the Korea Meteorological Administration. The data collected from multiple measurement stations spread across the entire nation were averaged to obtain national air pollution and meteorological data. Any missing values, if present, from the measurement stations were excluded during the averaging. Data regarding the hazard index of the daily pollen concentration from oaks, pines, and grasses were obtained from the Korea Meteorological Administration. This index was designed to forecast pollen concentrations based on meteorological and environmental factors (see Additional file 1: Table S1 for a detailed description). Weekly numbers of influenza and Middle East respiratory syndrome (MERS) patients were collected by the Korea Centers for Disease Control and Prevention. Patients with influenza were defined as those diagnosed with influenza (ICD-10 codes J10.0–J11.8) or those who had an influenza-like illness (ILI). ILI is defined by WHO as a respiratory infection with onset within the past ten days and a fever of ≥ 38 °C and cough or sore throat. Daily and regional data were averaged and converted into weekly national data for South Korea.

### Prediction of patients with asthma using environmental factors based on deep learning algorithms

To model the relationship between the number of patients with asthma (outpatients or ER patients) and environmental factors, we used 18 environmental factors and four potential confounders as input data. The 18 environmental factors included six air pollutant concentrations (CO, NO_2_, O_3_, PM_10_, PM_2.5_, and SO_2_), three pollen concentrations (pollen from oaks, pines, and grasses), eight meteorological conditions (mean temperature, minimum temperature, maximum temperature, diurnal temperature range, humidity, precipitation, solar radiation, and wind speed), and the number of patients with influenza. The four confounders were the week of the year, date, number of holidays per week, and number of patients with MERS. The week of the year is an indicator of where a particular week falls numerically within a year. The first week (week 1) of the year is defined as the week containing the first Wednesday of the year. For each year from 2015 to 2019, the week of the year is numbered from 1 to 52. The date is a number that incidates a specific point in time, and it is calculated as the number of days that have passed since January 1, 1970. These were used to model the confounding effects of seasonality, long-term trends, holidays, and the 2015 MERS outbreak. All input factors were preprocessed with minimum–maximum normalization before modeling.

We used the RNN, LSTM, and GRU as the deep learning algorithms. The model consisted of four layers: an input layer, two hidden layers, and an output layer. Recurrent cells were used only in the input layer, whereas simple, fully connected neural network cells without recurrent connections were used in the hidden and output layers to simplify the model (see Additional file 1: Figure S1 for the topology). Dropout techniques were applied to all layers to prevent overfitting [19, 20]. The optimum model size and dropout rates were selected empirically by finding the best model among various candidates (see Additional file 1: Table S2 for the hyperparameter candidates). We used walk-forward expanding window cross-validation, where data from 2015–2016, 2015–2017, and 2015–2018 were used for training and data from 2017, 2018, and 2019 were used for testing, respectively. Walk-forward cross-validation is a well-known validation method for time-series data to remove the possibility of prediction leakage [21, 22]. The length of time steps in RNN, LSTM, and GRU was set as 5 weeks to model the long-term lagged effects of environmental factors on asthma, and the training learning rate was set as 0.004. During training, the mean squared error (MSE) for the test set was monitored, and training was stopped when the observed MSE did not improve after 50 epochs. The modeling and training were implemented using the Python packages “keras” and “tensorflow” [23, 24].

### Comparison with conventional modeling methods

We compared the R^2^ values of the neural network models with those of the GLM, GAM, RF, and GBM. The input and output variables used for modeling were identical to those used for the deep learning algorithms. The GLM and GAM were fitted using the maximum likelihood method under the assumption of a quasi-Poisson distribution. For the modeling and training of RF and GBM, the “sklearn” package of Python was used. The model hyperparameters for RF and GBM were optimized using “best_estimator_” of the “Grid method for the model’s trainingSearchCV” function (see Additional file 1: Table S3 for the candidates) [25].

### Permutation feature importance

After modeling, we evaluated the importance of all input features in the final model based on the permutation feature importance method [26], where feature importance is defined as the increase in the MSE when the values of a single feature are temporally shuffled. This method helps identify features with high contributions in predicting the output. In addition, we define the interaction between the two features as follows:

1 $$I=F{I}_{AB}-F{I}_{A}-F{I}_{B}$$

here, I is the interaction between two features, A and B, FI_AB_ is the increase in MSE with both A and B shuffled, FI_A_ is the increase in MSE with A shuffled, and FI_B_ is the increase in MSE with B shuffled.

## Results

### General analysis of the number of patients with asthma

**Fig. 1:**
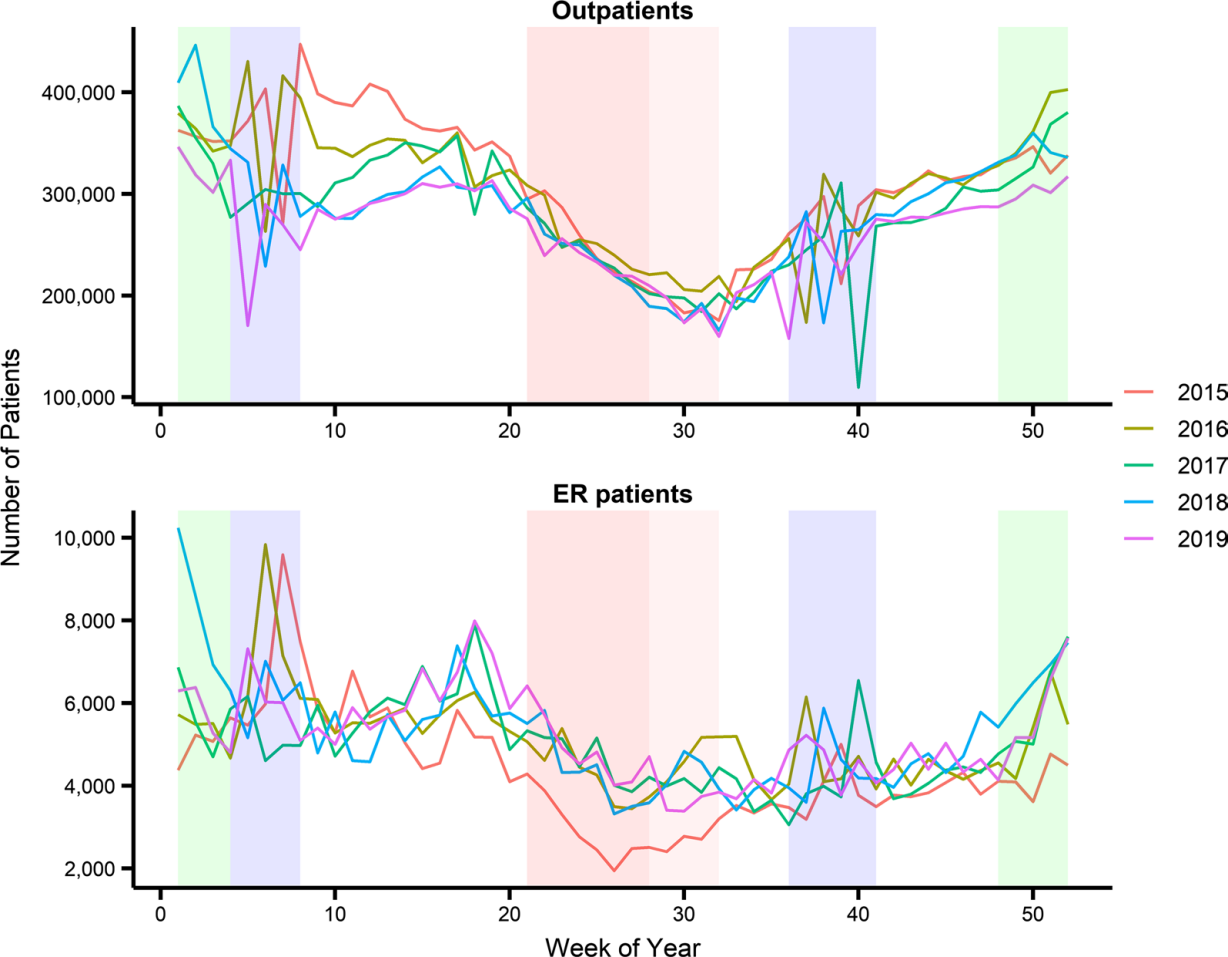
The number of outpatients and ER patients with asthma in South Korea each year from 2015 to 2019. The areas shaded in green, red, and blue highlighted the weeks when the numbers of influenza patients, patients with MERS, and holidays surged, respectively. The peaks in the green and blue periods correspond to the peaks in the number of influenza patients and the number of holidays in a week. The smaller number of ER visits during the red period in 2015 compared to 2016–2019 can be attributed to the MERS outbreak that occurred in 2015

Figure 1 shows the weekly numbers of outpatients and ER patients with asthma in South Korea for each year from 2015 to 2019 (see Additional file 1: Figure S2 for the 5-year curves and Additional file 1: Table S4 for descriptive statistics). From 2015 to 2019, the number of outpatients with asthma decreased, whereas the number of ER patients with asthma increased. The number of patients with asthma showed seasonal variability (Fig. 1), with the lowest in summer (July and August) and the highest in winter (December and January) and spring (March and April). Additional file 1: Figures S3-S8 show the time-series curves of various independent variables, and Additional file 1: Tables S5 and S6 show the descriptive statistics for air pollutant concentrations and climate conditions in South Korea during the study period.

The areas shaded in green in Fig. 1 (between Weeks 1 and 4, and between Weeks 48 and 52) show the weeks when the number of influenza patients surged, especially in 2018 (see Additional file 1: Figure S3 to find the surge in influenza patients). As shown in the figure, the number of patients with asthma increased during this period. The areas shaded in blue show the weeks in which the two biggest holidays in Korea, Lunar New Year’s Day (between Weeks 4 and 8) and Korean Thanksgiving Day (between Weeks 36 and 41), were located. During the holidays, the number of outpatients shows downward spikes due to the holiday clinic closures. In contrast, the number of ER patients shows upward spikes due to the “balloon effect” of the holiday clinic closures. This study used the number of holidays per week (Additional file 1: Figure S3) to model the confounding effects of holidays. The area shaded in darker red in Fig. 1 (between weeks 21 and 28) represents the weeks in 2015 when the MERS outbreak occurred in South Korea (see Additional file 1: Figure S3 for the number of patients with MERS). MERS is one of the 16 diseases classified as a “Class 1 infectious disease,” a term used for diseases of significant health importance owing to its high mortality rates; it was the only Class 1 infectious disease that caught public attention in South Korea during the study period. The area in lighter red (between weeks 28 and 32) shows the weeks between the last identification of patients with MERS and South Korea’s official declaration of a “de facto end” to MERS (July 28, 2015). During this period, the number of ER visits in many tertiary hospitals where patients with MERS were admitted was significantly reduced in 2015 because people were afraid of being infected. Since the MERS outbreak only occurred in 2015, the number of ER visits between weeks 21 and 32 in 2015 was apparently smaller than those during 2016–2019 as shown in Fig. 1.

This is a confounding effect of Class 1 infectious diseases, such as MERS, and we attempted to model this effect using the number of patients as an independent variable. Note that there is a four-week lag between the last identification of the patient with MERS (Week 28) and getting back to normal with the declaration of “de facto end” (Week 32). LSTM and GRU are suitable for modeling such long lags using their long-term memory.

### Modeling patients with asthma based on deep learning algorithms

**Fig. 2:**
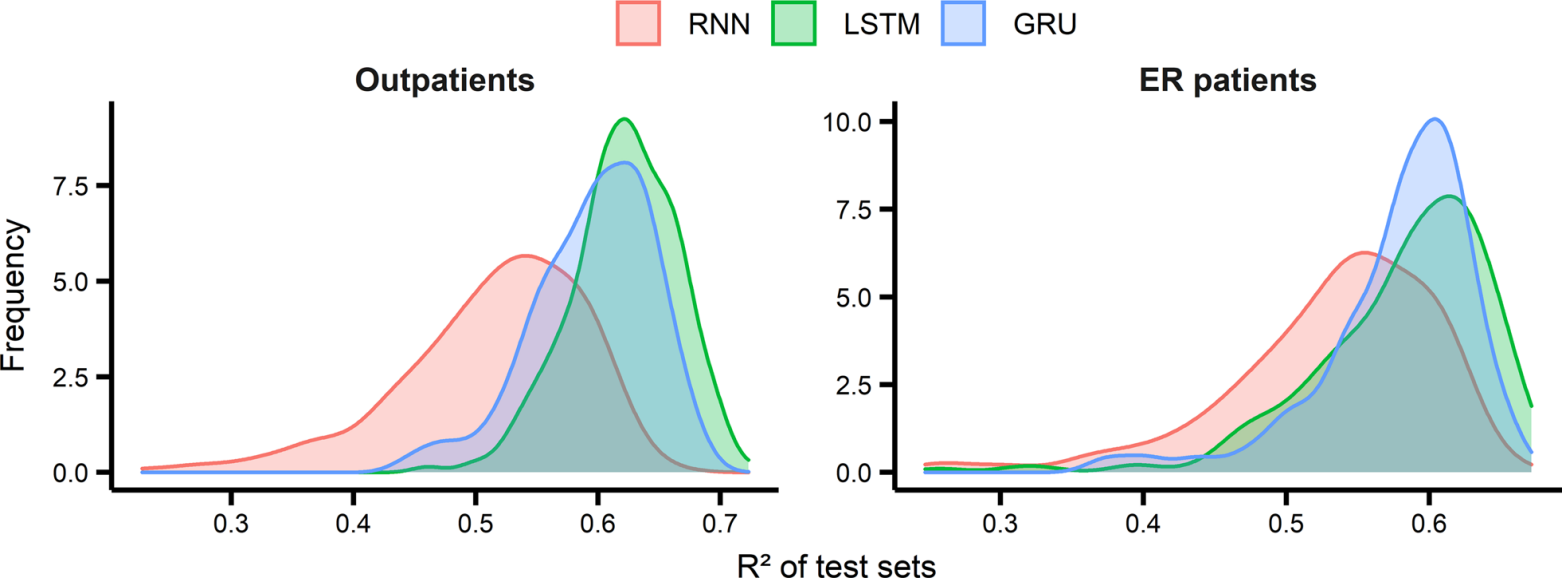
Performance (R^2^) histograms of 216 RNN, LSTM, and GRU models

To determine the best model for predicting the number of patients with asthma, we generated and trained 648 models of RNN, LSTM, and GRU (216 models each) and evaluated them with R^2^ based on a walk-forward cross-validation framework. Figure 2 shows the R^2^ histograms of 216 RNN, LSTM, and GRU models. The LSTM and GRU models performed better than the RNN models in predicting the number of patients with asthma. This may be due to the internal gates that solve the vanishing gradient problem in the RNN. Our results show that, in general, LSTM models perform slightly better than GRU models. This may be due to the higher number of gates in the LSTM model than in the GRU model (three in LSTM and two in GRU), providing more flexibility in modeling.

Additional file 1: Figure S9 shows the performance (R^2^) scatter plot for the 648 RNN, LSTM, and GRU models. The top 10% of the models are located in Area 1. Additional file 1: Table S7 lists all hyperparameter values of the models in Area 1. For the final model, we selected the model that provided the best average performance for outpatients and ER patients (R^2^ values of 0.723 for outpatients and 0.650 for ER patients).

### Performance comparison of asthma patient predictive models

Table 1 compares the performances of the final RNN, LSTM, and GRU models with those of conventional algorithms. The hyperparameters used in the final model are listed in Additional file 1: Table S7. Based on our results, LSTM was the best model for outpatients and ER patients, with R^2^ of 0.723 and 0.650, respectively. This algorithm performed better than the other algorithms investigated in this study. The ensemble-based models (RF and GBM) performed the worst for both outpatients and ER patients (R^2^ of − 0.321–0.583), whereas the regression-based models (GLM and GAM) ranked in the middle (R^2^ of 0.631–0.706).

**Table 1:** Performance (R^2^) comparison among various algorithms for training and test sets

Model	Training		Test		Gap (Training–Test)	
	Outpatients	ER patients	Outpatients	ER patients	Outpatients	ER patients
GLM	0.870	0.792	0.672	0.637	0.198	0.155
GAM	0.943	0.888	0.706	0.631	0.237	0.257
RF	0.857	0.508	0.540	-0.321	0.317	0.829
GBM	0.957	0.907	0.583	-0.128	0.374	1.035
RNN	0.894	0.803	0.625	0.606	0.269	0.197
LSTM	0.886	0.861	0.723	0.650	0.163	0.211
GRU	0.905	0.827	0.673	0.651	0.232	0.176

The R^2^ gap between the training and test sets was the smallest for the GLM, indicating the least overfitting among all algorithms. This is likely the result of linear modeling, which has a lower chance of overfitting than nonlinear modeling. The R^2^ gap was most significant for RF and GBM, indicating considerable overfitting. The gaps for RNN, LSTM, and GRU were smaller than those for GAM, RF, and GBM, despite the complexity and flexibility of the models. This results from the dropout and early stopping techniques implemented in training.

### Feature importance analysis

**Fig. 3:**
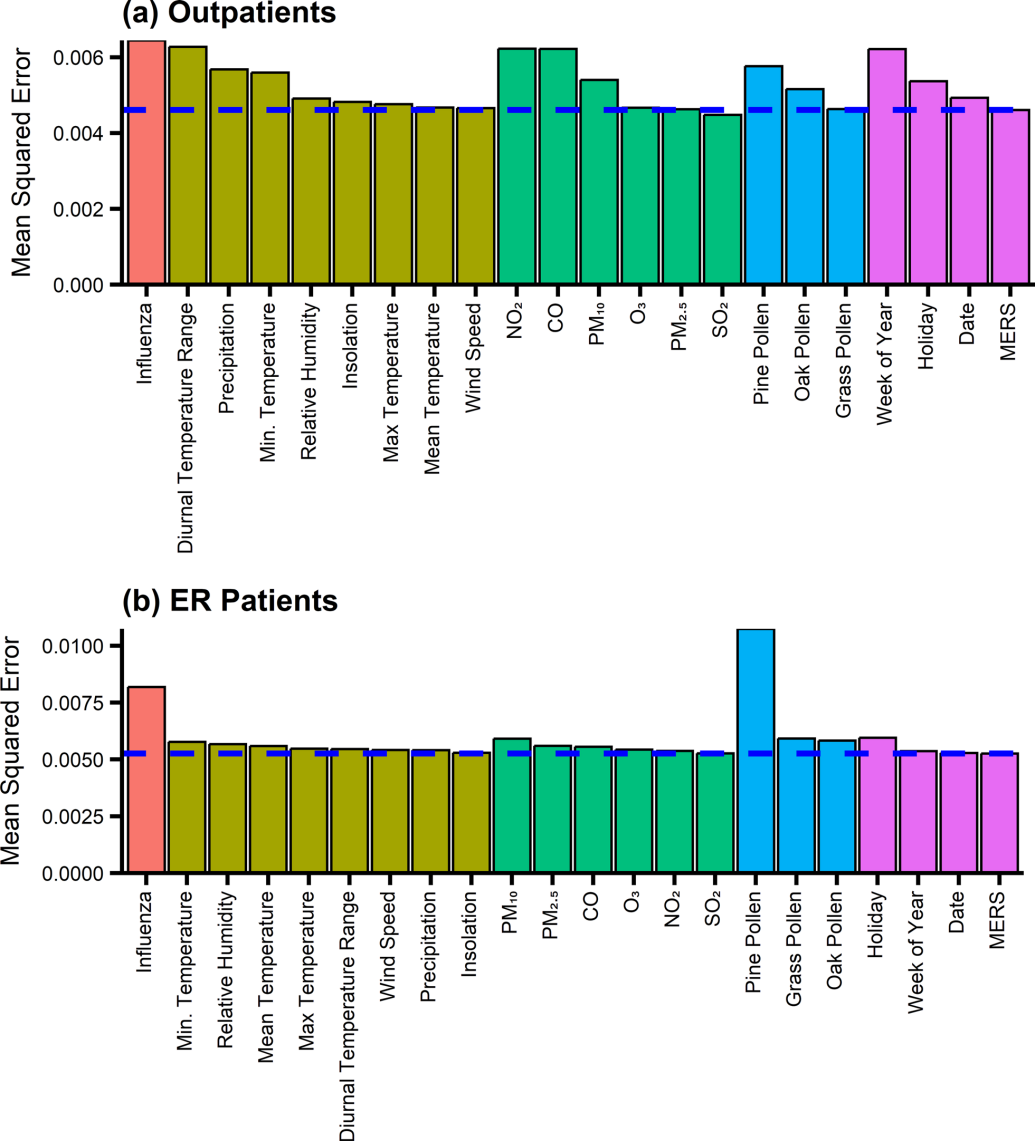
Feature importance of (**a**) outpatients and (**b**) ER patients

Figure 3 shows the results of the feature importance analysis for outpatients and patients in the ER. In Fig. 3, the blue dashed line shows the model’s baseline MSE, and any feature that yielded a higher MSE than the baseline after shuffling was considered significant (the higher the value, the more important it is). Influenza was one of the most important factors for outpatients and ER patients among the various environmental factors. Influenza can cause airway swelling, trigger asthma attacks, and exacerbate symptoms [27]. Temperature substantially impacted the number of outpatient visits and had little effect on the number of ER visits. This can be interpreted as the temperature being associated more with the gradual development of asthma than the acute development of asthma, which causes patients to visit non-emergency care facilities. Among four temperature-related factors (mean, maximum, minimum, and diurnal temperature range), the diurnal temperature range and minimum temperature were the two most critical factors affecting outpatients with asthma. Air pollutants such as NO_2_, CO, and PM_10_ had a significant impact on outpatients while they had little impact on ER patients. This may indicate that asthma exacerbation attributable to NO_2_, CO, and PM_10_ is not severe enough for patients to visit ER. Pine pollen had a substantial impact on the number of ER patients with asthma, whereas it had a relatively smaller impact on the number of outpatients with asthma. The association between pine pollen and ER patients with asthma can be observed in Additional file 1: Figure S8 during the pine pollen season (between weeks 16 and 20). The week of the year and the number of holidays per week were also important in modeling the seasonal variability and confounding effect of holiday clinic closure, respectively. Additional file 1: Figure S10 shows the two-dimensional feature importance analysis. Our result indicates that the simultaneous exposure to both NO_2_ and CO has a synergic effect on asthma exacerbation. However, there is no significant interaction effect among other environmental factors.

## Discussion

The climate is known for impacting asthma directly through the airway response to climate or indirectly through altered exposure to air pollutants, allergens, and pathogens [28]. For example, high temperatures increase the ambient ozone through a photochemical reaction [29]. In addition, abrupt changes in weather and an extensive diurnal temperature range can increase the risk of asthma by affecting inflammatory responses and immunity [30–32]. In this study, we examined the association between outpatients with asthma and climate, and we showed that diurnal temperature range and minimum temperature are important factors in modeling. This result agrees with the results of previous studies. Chen et al. showed that both low and high temperatures were associated with an increased risk of asthma, whereas the majority of the burden was attributable to moderate cold exposures [2]. Xu et al. and Kim et al. studied the relationship between the diurnal temperature difference and asthma [33, 34].

Air pollutants are associated with asthma through both direct and indirect mechanisms [35]. The infiltration of air pollutants can directly trigger inflammation and increase oxidative stress, which may lead to cell and tissue damage in airways [36–38]. Air pollutants can also be involved in indirect mechanisms interacting with inhaled pathogens and allergens, thereby increasing the risk of infections and allergic reactions [35]. The influence of NO_2_ and CO on asthma patients was observed in this study, which agrees with a previous study demonstrating the impact of exposure to traffic emissions such as NO_2_ and CO on asthma [39]. Additionally, PMs may act as a container for allergens and pathogens such as pollen, fungal spores, and viruses, delivering them deep into the airways [40, 41]. In this study, the risk of asthma exacerbation with PM_10_ was higher than that with PM_2.5_, which is consistent with the results of a previous study by Tecer et al. [42].

Influenza infection can trigger an immune response by releasing cytokines and increasing susceptibility to asthma [27, 43]. Several studies have reported that influenza is associated with asthma exacerbation in adults [10, 44, 45].

This study had a few limitations. First, public environmental data was measured at official stations, instead of personal exposure data. This may have resulted in the underestimation of the actual impact of exposure to asthma-exacerbating environments. Second, demographic factors (such as age, gender, and sex) and socioeconomic conditions (such as occupation, income level, and educational attainment) were not considered in this study because such information was unavailable. Additional studies are warranted to consider such factors in modeling to increase the accuracy.

## Conclusions

This study is the first to analyze the association between outpatients and ER patients with asthma and 18 environmental factors, including air pollutants, weather conditions, pollen, and influenza, in South Korea. Additionally, it proved the relative and quantitative importance of all 18 factors in terms of asthma exacerbation. Models with various hyperparameter values were evaluated to optimize the deep learning algorithm. With the optimal hyperparameters, we found that LSTM was the best model for predicting patients with asthma among the eight algorithms studied. It can model nonlinear lagged relationships with interactions between features without causing multicollinearity and overfitting problems. From feature importance analysis, we found out that influenza and pine pollen were the two most important factors exacerbating asthma in outpatients and ER patients.

### Supplementary Information

**Additional file 1. Table S1.** Information on pollen. **Table S2.** Hyperparameter candidates for the neural network models. **Table S3.** Hyperparameters considered for RF and GBM. **Table S4.** Descriptive statistics for the number of asthma patients in South Korea between 2015 and 2019. **Table S5.** Descriptive statistics for the air pollutant concentrations in South Korea between 2015 and 2019. **Table S6.** Descriptive statistics for the climate conditions in South Korea between 2015 and 2019. **Table S7.** The hyperparameter values of models in Area 1 of Figure S9 and the hyperparameter values selected for the final model. **Figure S1.** Topology of deep learning algorithms. **Figure S2.** The number of asthma outpatients and ER patients in South Korea between 2015 and 2019. The black line shows the linear regression fit of the patient data. **Figure S3.** The numbers of asthma patients, influenza patients, MERS patients, holidays in a week in South Korea in each year from 2015 to 2019. The areas shaded in green, red, and blue highlighted the weeks, when the numbers of influenza patients, MERS patients and holidays surged, respectively. **Figure S4.** The number of asthma patients and pollutant concentrations (CO, O_3_, and SO_2_) in South Korea in each year from 2015 to 2019. **Figure S5.** The number of asthma patients and pollutant concentrations (NO_2_, PM_10_, and PM_2·5_) in South Korea in each year from 2015 to 2019. **Figure S6.** The number of asthma patients, temperature, and relative humidity in South Korea in each year from 2015 to 2019. **Figure S7.** The number of asthma patients, precipitation, wind speed, and insolation in South Korea in each year from 2015 to 2019. **Figure S8.** The number of asthma patients and pollen hazard index in South Korea in each year from 2015 to 2019. **Figure S9.** Performance (R^2^) scatter plot of modeling outpatients (x axis) and ER patients (y axis) for 648 models of RNN, LSTM, and GRU. The red dashed lines indicate the 90 percentile of R^2^ for outpatients and ER patients. The four areas that were divided with the red dashed lines were denoted as Areas 1 to 4. **Figure S10.** Observed and predicted number of asthma patients for outpatient and ER patients.

## Data Availability

The datasets used and/or analyzed during the current study are available from the corresponding author on reasonable request.

## Abbreviations

GLM: Generalized linear model
GAM: Generalized additive model
DLNM: Distributed lag nonlinear model
RF: Random forest
GBM: Gradient boosting machine
RNN: Recurrent neural network
LSTM: Long short-term memory
GRU: Gated recurrent unit
PM: Particulate matter
ER: Emergency room
ILI: Influenza-like illness
MERS: Middle East respiratory syndrome
MSE: Mean squared error
R^2^: Coefficient of determination
