# Supplementary material for: Prediction of the number of asthma patients using environmental factors based on deep learning algorithms
Source: Respir Res. 2023 Dec 1;24:302. doi: 10.1186/s12931-023-02616-x (PMC10693131; doi:10.1186/s12931-023-02616-x)
Supplement: Supplementary file 1 — Additional file 1. Table S1. Information on pollen. Table S2. Hyperparameter candidates for the neural network models. Table S3. Hyperparameters considered for RF and GBM. Table S4. Descriptive statistics for the number of asthma patients in South Korea between 2015 and 2019. Table S5. Descriptive statistics for the air pollutant concentrations in South Korea between 2015 and 2019. Table S6. Descriptive statistics for the climate conditions in South Korea between 2015 and 2019. Table S7. The hyperparameter values of models in Area 1 of Figure S9 and the hyperparameter values selected for the final model. Figure S1. Topology of deep learning algorithms. Figure S2. The number of asthma outpatients and ER patients in South Korea between 2015 and 2019. The black line shows the linear regression fit of the patient data. Figure S3. The numbers of asthma patients, influenza patients, MERS patients, holidays in a week in South Korea in each year from 2015 to 2019. The areas shaded in green, red, and blue highlighted the weeks, when the numbers of influenza patients, MERS patients and holidays surged, respectively. Figure S4. The number of asthma patients and pollutant concentrations (CO, O3, and SO2) in South Korea in each year from 2015 to 2019. Figure S5. The number of asthma patients and pollutant concentrations (NO2, PM10, and PM2·5) in South Korea in each year from 2015 to 2019. Figure S6. The number of asthma patients, temperature, and relative humidity in South Korea in each year from 2015 to 2019. Figure S7. The number of asthma patients, precipitation, wind speed, and insolation in South Korea in each year from 2015 to 2019. Figure S8. The number of asthma patients and pollen hazard index in South Korea in each year from 2015 to 2019. Figure S9. Performance (R2) scatter plot of modeling outpatients (x axis) and ER patients (y axis) for 648 models of RNN, LSTM, and GRU. The red dashed lines indicate the 90 percentile of R2 for outpatients and ER patients. The four a [file 12931_2023_2616_MOESM1_ESM.docx]

**Prediction of the Number of Asthma Patients Using Environmental Factors Based on Deep Learning Algorithms**

Hyemin Hwang^a^, Jae-Hyuk Jang^b^, Eunyoung Lee^c^, Hae-Sim Park^b^ and Jae Young Lee^d,*^

^a^Environmental Engineering Department, Ajou University, Suwon 16499, Korea; hhm8866@ajou.ac.kr

^b^Department of Allergy and Clinical Immunology, Ajou University School of Medicine, Suwon 16499, Korea; silverlining999@ajou.ac.kr (J.-H. Jang); hspark@ajou.ac.kr (H.-S. Park)

^c^Department of Neurology, McGovern Medical School, The University of Texas Health Science Center at Houston, Houston, TX 77030, USA; e.angie.lee@gmail.com

^d^Environmental and Safety Engineering Department, Ajou University, Suwon 16499, Korea; jaeylee@ajou.ac.kr

* Correspondence to Prof. Jae Young Lee; 206, World cup-ro, Yeongtong-gu, Suwon 16499, Korea; +82-31-219-2404; jaeylee@ajou.ac.kr

**Table S1.** Information on pollen

|  | **Oak** | **Pine** | **Grass** |
| --- | --- | --- | --- |
| Scientific name | *Q. variavilis BI.* | *Pinus densiflora Siebold & Zucc.* | *Zoysia japonica Steud.* |
| Identification name | Quercus | Pinaceae | Poaceae |
| Size | 30~45μm | 45~70μm | 22~100μm |

**
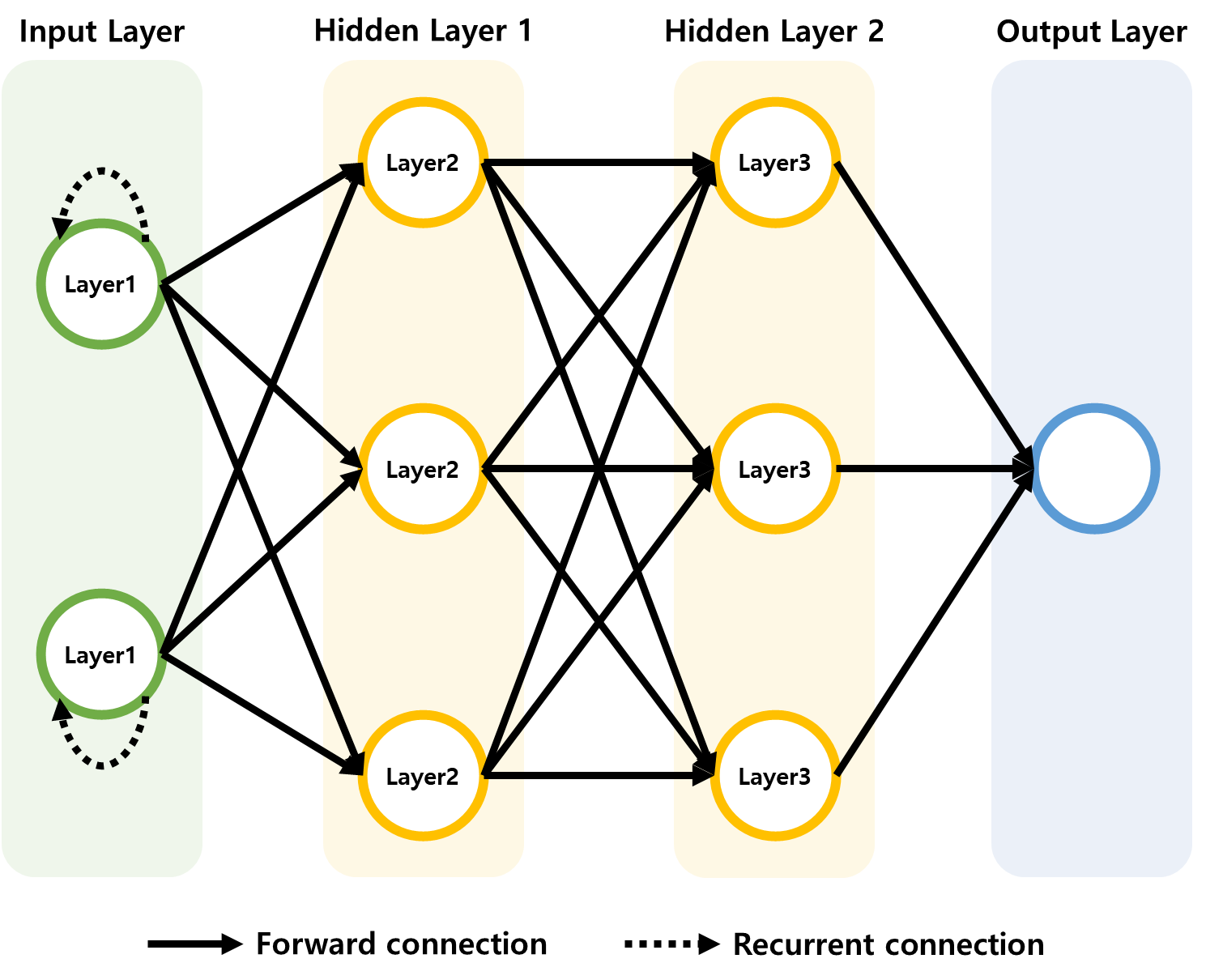
**

**Figure S1.** Topology of deep learning algorithms

**Table S2.** Hyperparameter candidates for the neural network models.

| **Algorithm** | **Number of units** | | | **Dropout rate** | | |
| --- | --- | --- | --- | --- | --- | --- |
|  | **Layer 1** | **Layer 2** | **Layer 3** | **Input** | **Feedforward** | **Recurrent** |
| RNN  LSTM  GRU | 64, 128 | 8, 16 | 8, 16 | 0, 0.1, 0.3 | 0, 0.1, 0.3 | 0, 0.1, 0.3 |

**Table S3.** Hyperparameters considered for RF and GBM.

| **Hyperparameter** | **Value** |
| --- | --- |
| The number of trees | 100, 200, 300 |
| The maximum depth of the tree | 4, 6, 8, 10, 12 |
| The minimum number of samples required to split an internal node | 6, 8, 10, 12, 14 |
| The minimum number of samples required to be at a leaf node | 8, 16, 20, 24 |

**
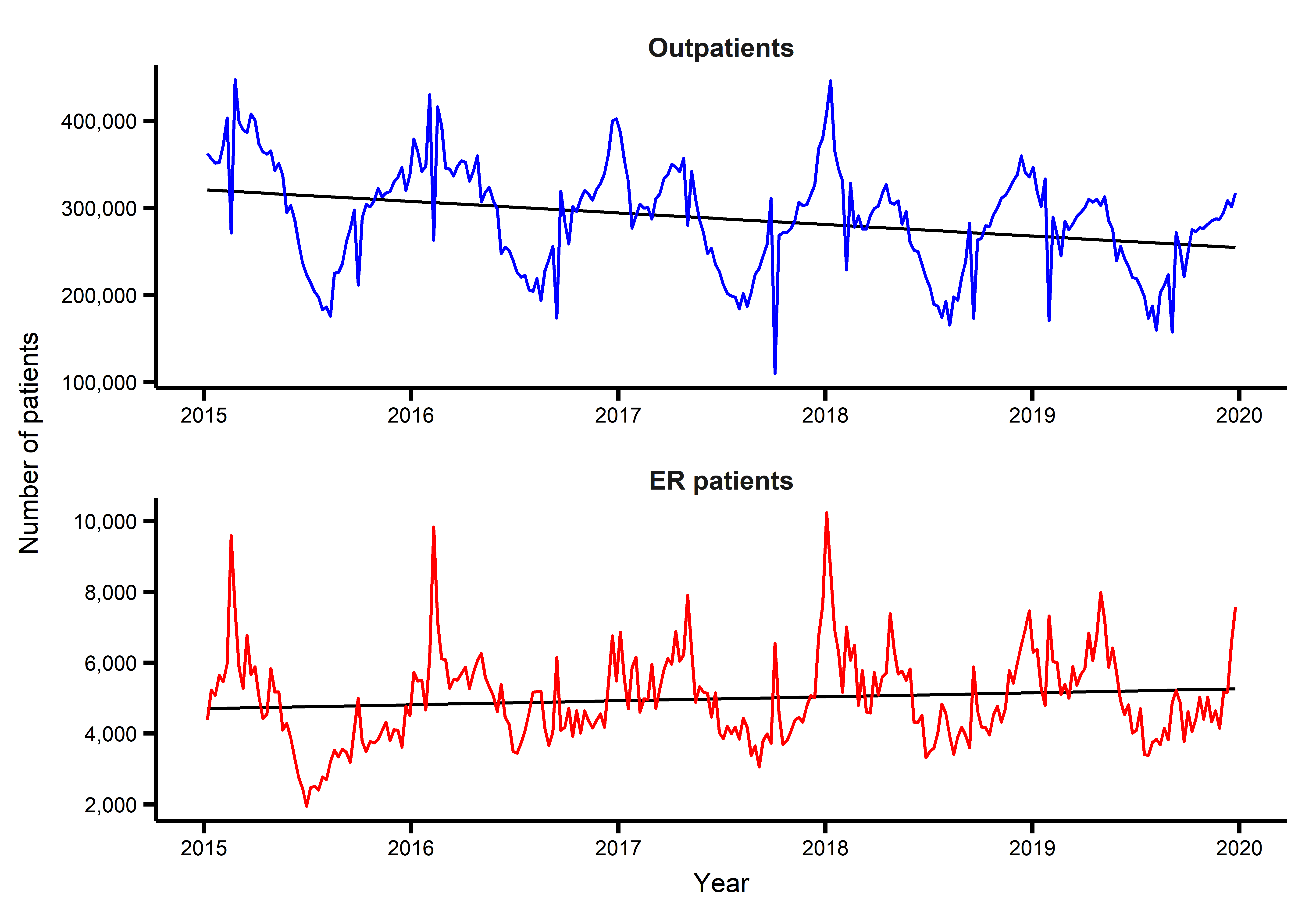
**

**Figure S2.** The number of asthma outpatients and ER patients in South Korea between 2015 and 2019. The black line shows the linear regression fit of the patient data.

**Table S4.** Descriptive statistics for the number of asthma patients in South Korea between 2015 and 2019.

| **Patients** | **Year** | **Min.** | **25^th^ percentiles** | **Median** | **75^th^ percentiles** | **Max.** | **Mean** | **SD.** |
| --- | --- | --- | --- | --- | --- | --- | --- | --- |
| Outpatients | Average | 109,765 | 241,807 | 293,504 | 330,225 | 447,192 | 287,684 | 61,201 |
|  | 2015 | 175,468 | 260,621 | 318,002 | 357,855 | 447,192 | 308,407 | 66,944 |
|  | 2016 | 173,596 | 253,960 | 316,921 | 345,924 | 430,166 | 304,995 | 61,592 |
|  | 2017 | 109,765 | 242,422 | 286,913 | 318,800 | 386,703 | 281,174 | 58,163 |
|  | 2018 | 165,686 | 237,583 | 286,676 | 317,959 | 446,307 | 280,434 | 60,144 |
|  | 2019 | 157,698 | 230,543 | 276,171 | 296,306 | 346,310 | 263,408 | 46,112 |
| ER patients | Average | 1,945 | 4,096 | 4,810 | 5,766 | 10,245 | 4,984 | 1,265 |
|  | 2015 | 1,945 | 3,489 | 4,104 | 5,177 | 9,592 | 4,335 | 1,391 |
|  | 2016 | 3,447 | 4,244 | 5,178 | 5,616 | 9,838 | 5,097 | 1,089 |
|  | 2017 | 3,059 | 4,144 | 4,825 | 5,883 | 7,909 | 5,009 | 1,112 |
|  | 2018 | 3,319 | 4,286 | 4,963 | 5,908 | 10,245 | 5,279 | 1,371 |
|  | 2019 | 3,385 | 4,381 | 5,031 | 5,920 | 7,989 | 5,197 | 1,111 |

**
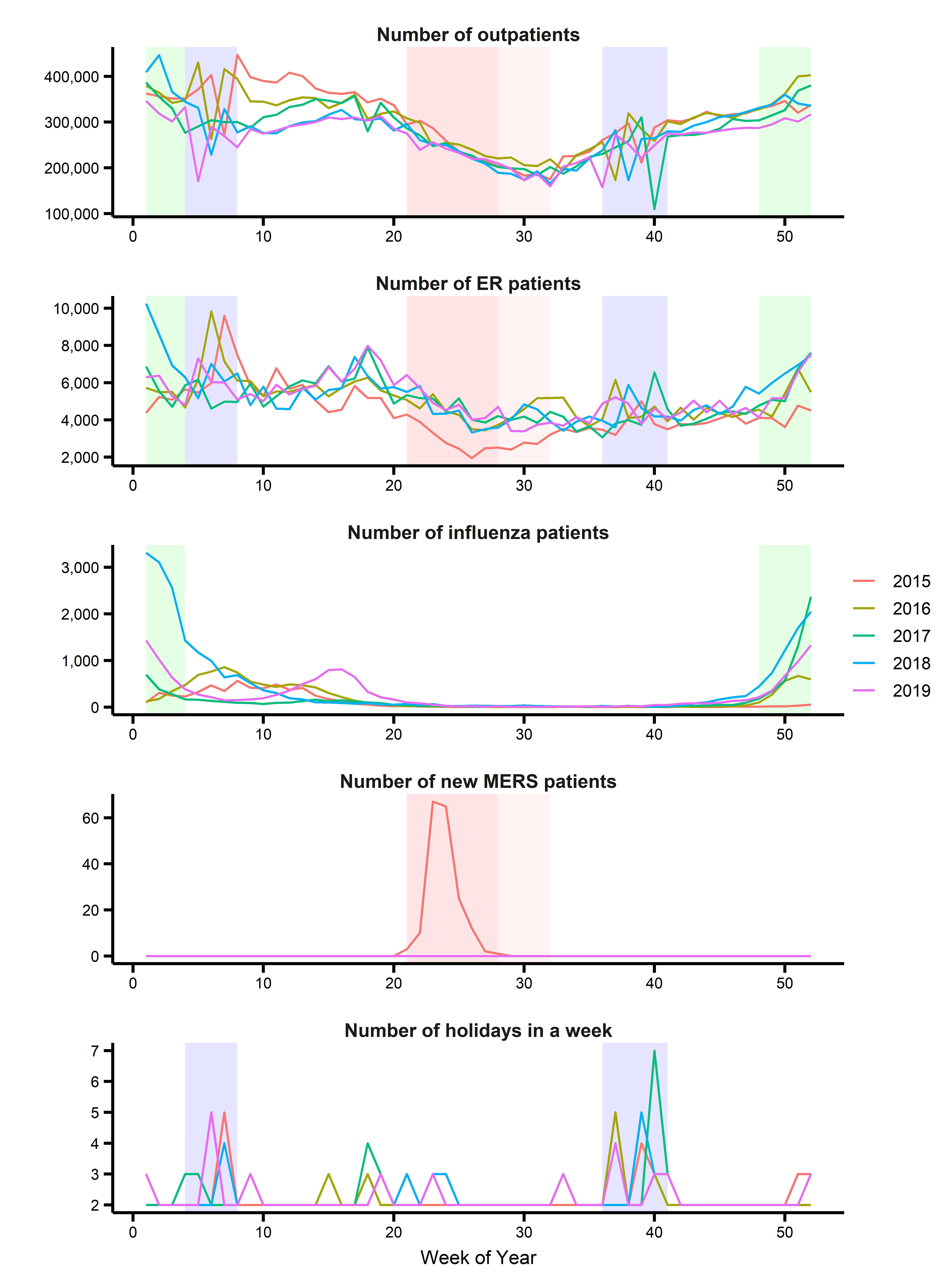
**

**Figure S3.** The numbers of asthma patients, influenza patients, MERS patients, holidays in a week in South Korea in each year from 2015 to 2019. The areas shaded in green, red, and blue highlighted the weeks, when the numbers of influenza patients, MERS patients and holidays surged, respectively.

**
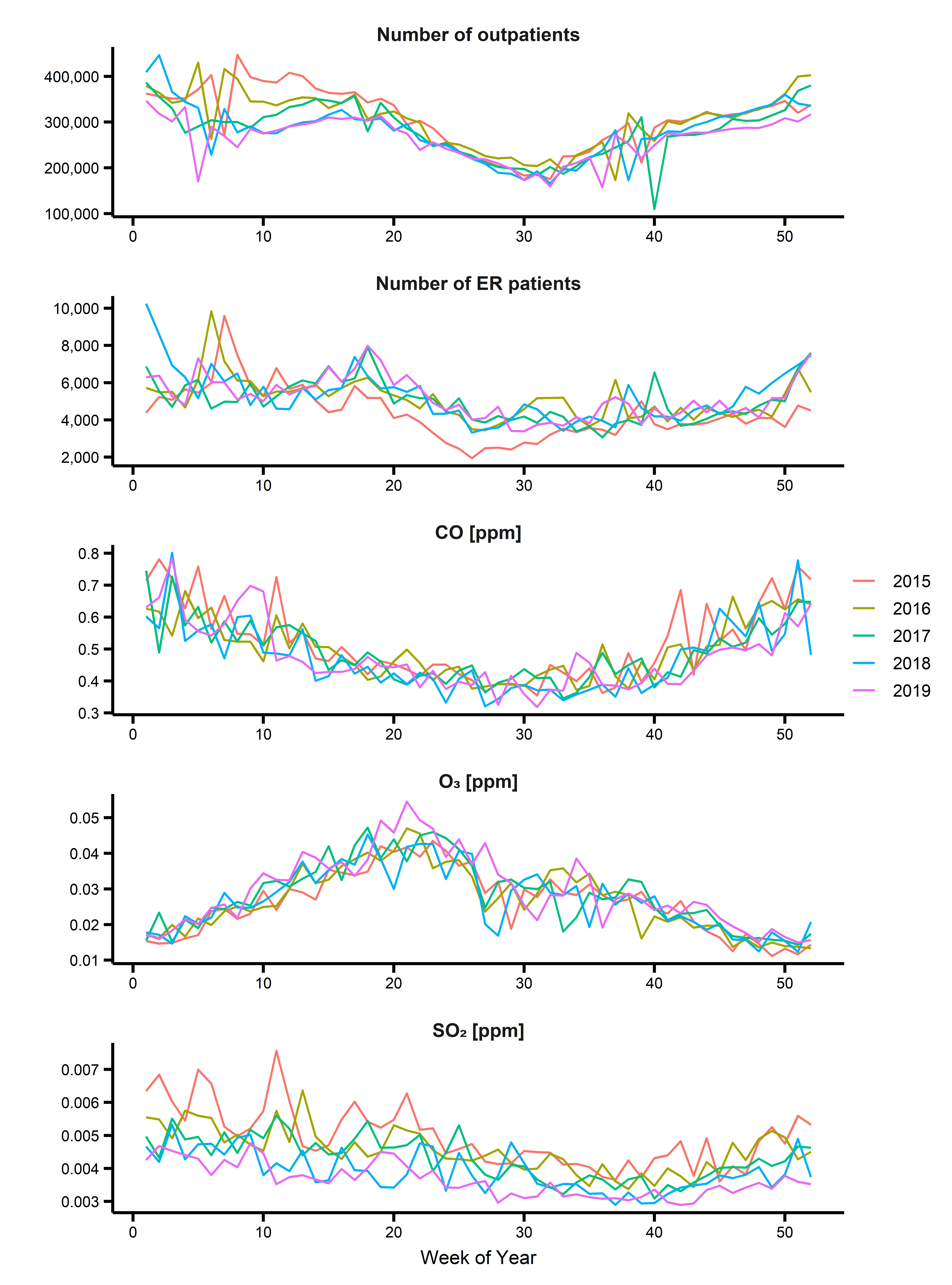
**

**Figure S4.** The number of asthma patients and pollutant concentrations (CO, O_3_, and SO_2_) in South Korea in each year from 2015 to 2019.

**
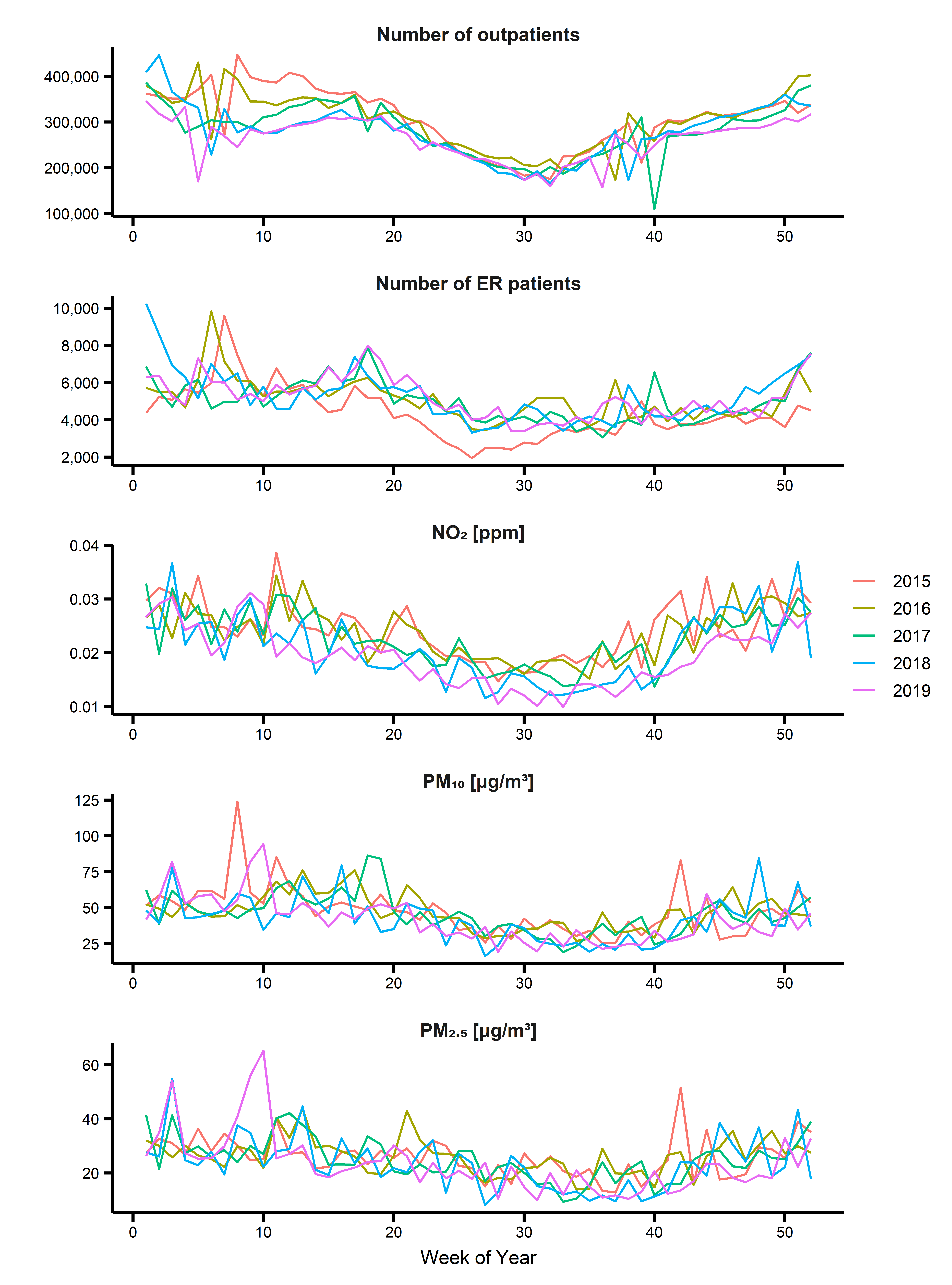
**

**Figure S5.** The number of asthma patients and pollutant concentrations (NO_2_, PM_10_, and PM_2.5_) in South Korea in each year from 2015 to 2019.

**
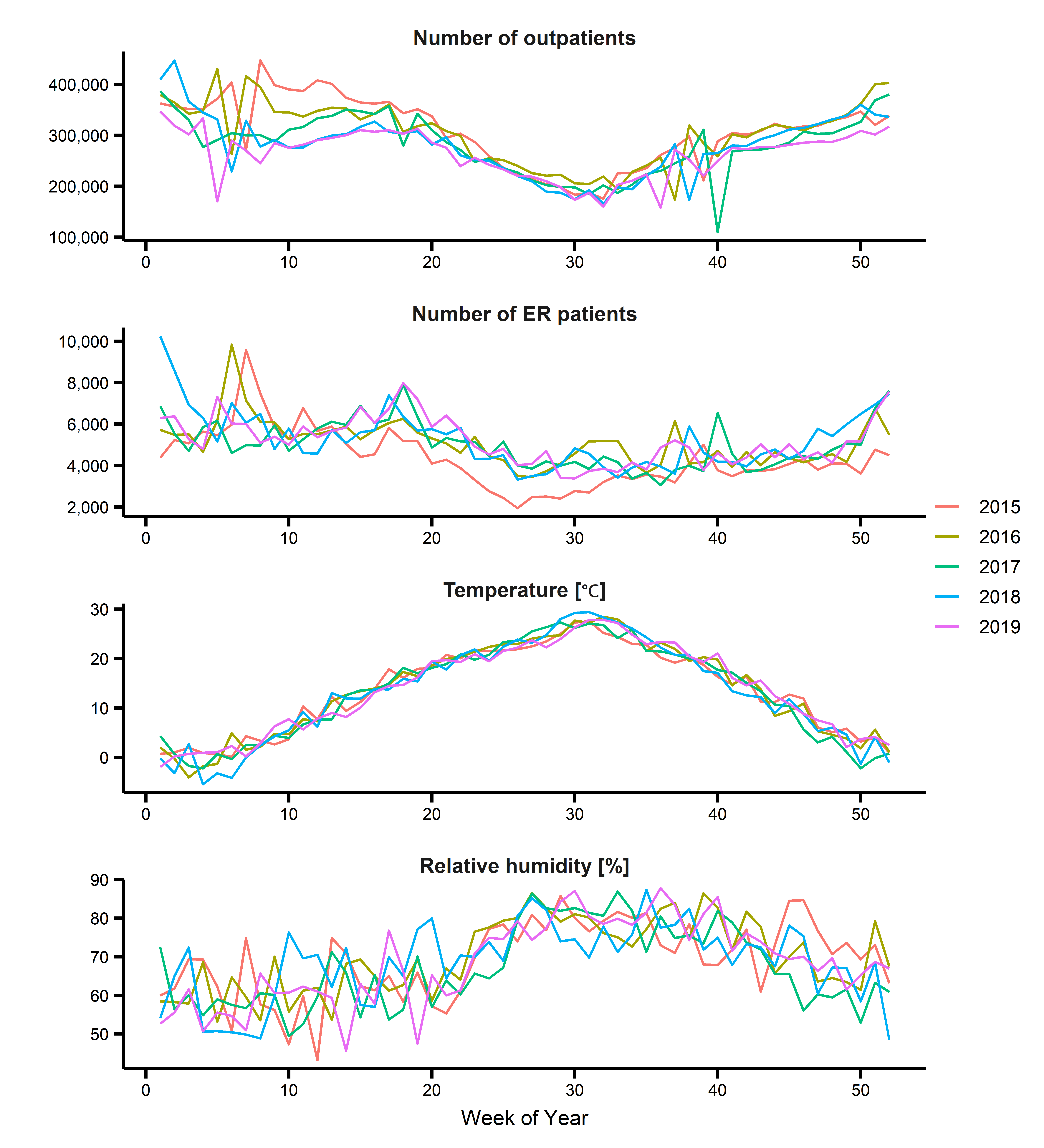
**

**Figure S6.** The number of asthma patients, temperature, and relative humidity in South Korea in each year from 2015 to 2019.

**
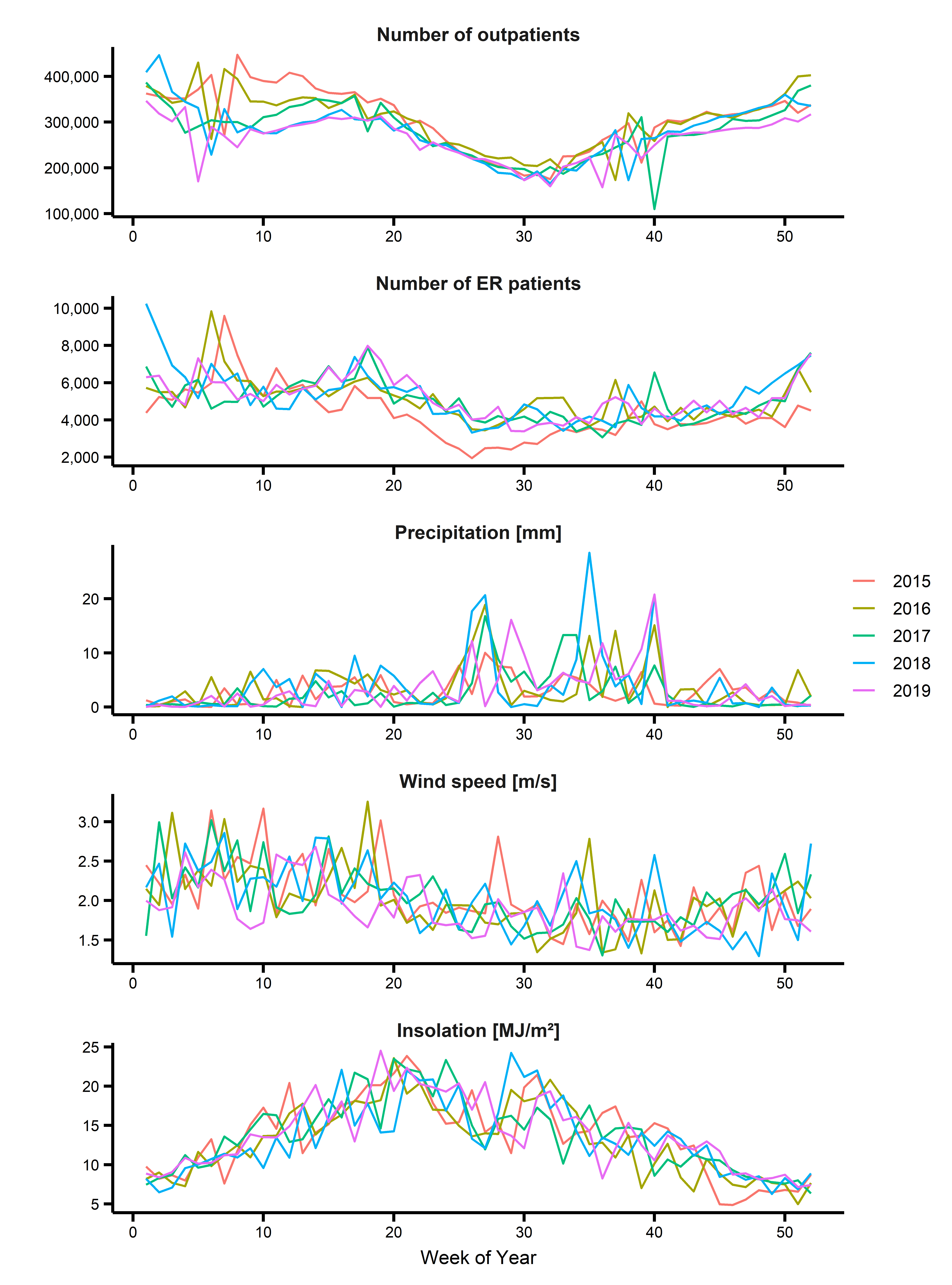
**

**Figure S7.** The number of asthma patients, precipitation, wind speed, and insolation in South Korea in each year from 2015 to 2019.

**
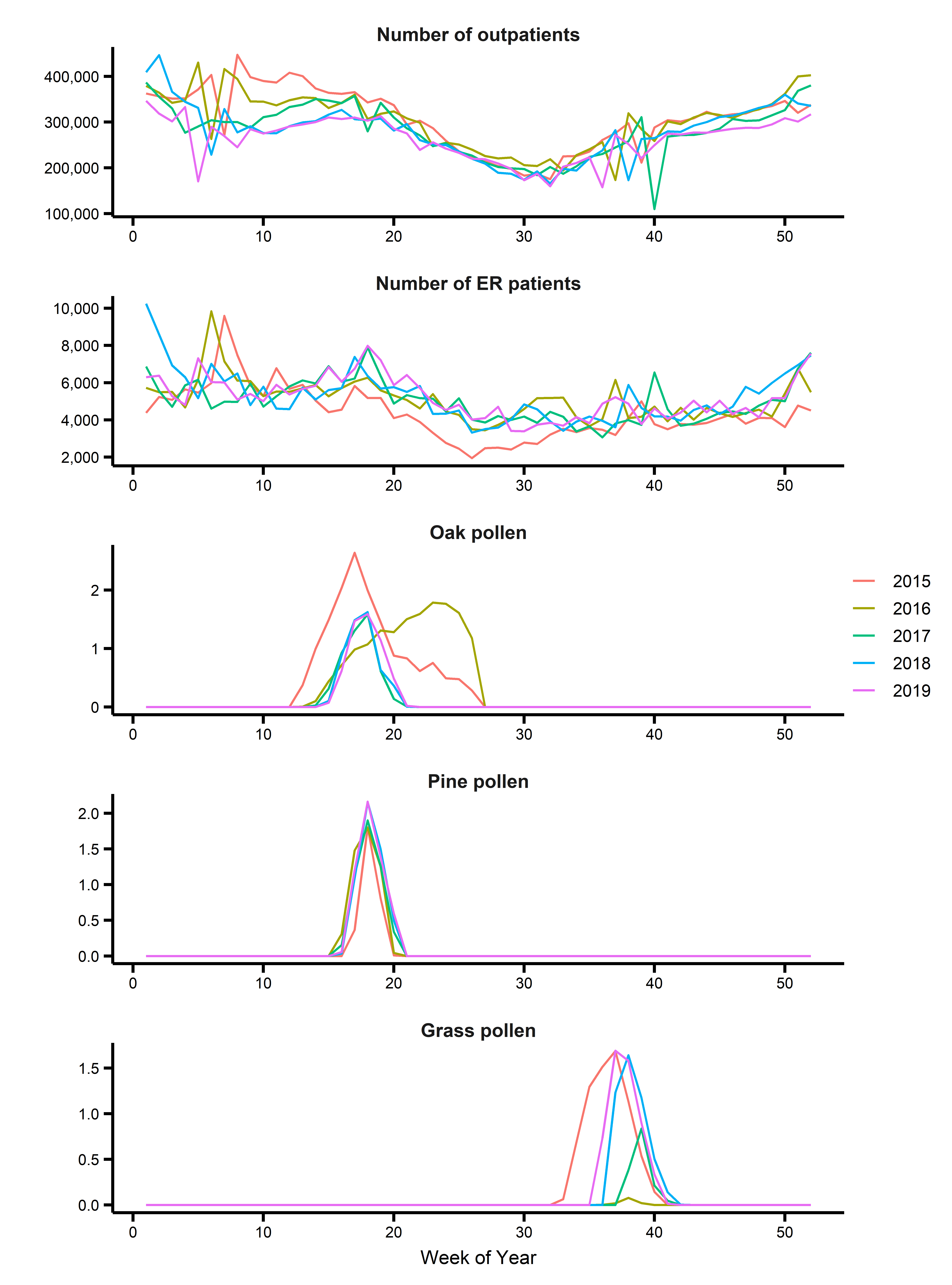
**

**Figure S8.** The number of asthma patients and pollen hazard index in South Korea in each year from 2015 to 2019.

**Table S5.** Descriptive statistics for the air pollutant concentrations in South Korea between 2015 and 2019.

| **Factors** | **Year** | **Min.** | **25^th^ percentiles** | **Median** | **75^th^ percentiles** | **Max.** | **Mean** | **SD.** |
| --- | --- | --- | --- | --- | --- | --- | --- | --- |
| CO  (ppm) | Average | 0.318 | 0.410 | 0.465 | 0.556 | 0.801 | 0.491 | 0.105 |
|  | 2015 | 0.355 | 0.421 | 0.479 | 0.626 | 0.781 | 0.521 | 0.123 |
|  | 2016 | 0.372 | 0.416 | 0.500 | 0.568 | 0.682 | 0.499 | 0.091 |
|  | 2017 | 0.345 | 0.415 | 0.478 | 0.546 | 0.745 | 0.490 | 0.091 |
|  | 2018 | 0.321 | 0.389 | 0.442 | 0.541 | 0.801 | 0.471 | 0.107 |
|  | 2019 | 0.318 | 0.393 | 0.449 | 0.522 | 0.782 | 0.477 | 0.103 |
| NO_2_  (ppm) | Average | 0.010 | 0.018 | 0.022 | 0.026 | 0.039 | 0.022 | 0.006 |
|  | 2015 | 0.015 | 0.020 | 0.024 | 0.028 | 0.039 | 0.024 | 0.005 |
|  | 2016 | 0.015 | 0.019 | 0.024 | 0.027 | 0.034 | 0.024 | 0.005 |
|  | 2017 | 0.014 | 0.019 | 0.022 | 0.026 | 0.033 | 0.023 | 0.005 |
|  | 2018 | 0.012 | 0.016 | 0.019 | 0.026 | 0.037 | 0.021 | 0.006 |
|  | 2019 | 0.010 | 0.015 | 0.019 | 0.023 | 0.031 | 0.019 | 0.006 |
| O_3_  (ppm) | Average | 0.011 | 0.020 | 0.027 | 0.034 | 0.054 | 0.028 | 0.009 |
|  | 2015 | 0.011 | 0.018 | 0.027 | 0.032 | 0.043 | 0.026 | 0.009 |
|  | 2016 | 0.013 | 0.020 | 0.025 | 0.035 | 0.047 | 0.027 | 0.009 |
|  | 2017 | 0.014 | 0.021 | 0.027 | 0.033 | 0.047 | 0.028 | 0.009 |
|  | 2018 | 0.012 | 0.020 | 0.027 | 0.033 | 0.045 | 0.027 | 0.009 |
|  | 2019 | 0.015 | 0.021 | 0.028 | 0.037 | 0.054 | 0.030 | 0.010 |
| SO_2_  (ppm) | Average | 0.003 | 0.004 | 0.004 | 0.005 | 0.008 | 0.004 | 0.001 |
|  | 2015 | 0.004 | 0.004 | 0.005 | 0.005 | 0.008 | 0.005 | 0.001 |
|  | 2016 | 0.003 | 0.004 | 0.005 | 0.005 | 0.006 | 0.005 | 0.001 |
|  | 2017 | 0.003 | 0.004 | 0.004 | 0.005 | 0.006 | 0.004 | 0.001 |
|  | 2018 | 0.003 | 0.003 | 0.004 | 0.004 | 0.005 | 0.004 | 0.001 |
|  | 2019 | 0.003 | 0.003 | 0.004 | 0.004 | 0.005 | 0.004 | 0.000 |
| PM_10_  (μg/m^3^) | Average | 16.4 | 33.8 | 43.6 | 53.0 | 123.9 | 44.7 | 15.2 |
|  | 2015 | 25.2 | 35.6 | 46.9 | 55.1 | 123.9 | 47.8 | 17.1 |
|  | 2016 | 26.8 | 38.7 | 46.1 | 54.0 | 76.3 | 47.0 | 12.1 |
|  | 2017 | 19.1 | 38.2 | 44.0 | 52.5 | 86.3 | 45.5 | 13.7 |
|  | 2018 | 16.4 | 30.7 | 40.3 | 48.2 | 84.5 | 41.6 | 15.7 |
|  | 2019 | 19.3 | 30.3 | 37.8 | 49.5 | 94.5 | 41.4 | 15.8 |
| PM_2.5_  (μg/m^3^) | Average | 8.2 | 19.4 | 24.0 | 28.8 | 65.3 | 24.8 | 8.7 |
|  | 2015 | 12.8 | 21.7 | 25.6 | 29.2 | 51.5 | 25.9 | 7.3 |
|  | 2016 | 13.9 | 21.6 | 26.5 | 29.8 | 43.7 | 26.2 | 6.7 |
|  | 2017 | 9.4 | 20.5 | 24.0 | 28.4 | 42.2 | 25.0 | 7.8 |
|  | 2018 | 8.2 | 16.8 | 22.6 | 27.9 | 54.8 | 23.4 | 9.8 |
|  | 2019 | 10.0 | 16.9 | 21.5 | 26.3 | 65.3 | 23.4 | 11.0 |

**Table S6.** Descriptive statistics for the climate conditions in South Korea between 2015 and 2019.

| **Factors** | **Year** | **Min.** | **25^th^ percentiles** | **Median** | **75^th^ percentiles** | **Max.** | **Avg.** | **SD.** |
| --- | --- | --- | --- | --- | --- | --- | --- | --- |
| Temperature  (℃) | Average | -5.4 | 4.7 | 13.9 | 21.4 | 29.4 | 13.4 | 9.2 |
|  | 2015 | 0.1 | 4.9 | 14.3 | 20.9 | 27.6 | 13.5 | 8.4 |
|  | 2016 | -4.0 | 4.8 | 14.3 | 21.6 | 28.4 | 13.6 | 9.3 |
|  | 2017 | -2.2 | 4.1 | 14.3 | 20.8 | 27.3 | 13.1 | 9.5 |
|  | 2018 | -5.4 | 5.2 | 13.2 | 21.1 | 29.4 | 13.1 | 9.9 |
|  | 2019 | -1.9 | 6.1 | 14.5 | 21.2 | 27.8 | 13.5 | 8.8 |
| Relative humidity  (%) | Average | 43.2 | 60.9 | 69.4 | 77.0 | 87.8 | 68.7 | 10.0 |
|  | 2015 | 43.2 | 61.7 | 70.8 | 77.0 | 85.8 | 69.3 | 9.8 |
|  | 2016 | 53.2 | 62.5 | 69.4 | 78.1 | 86.6 | 69.9 | 9.3 |
|  | 2017 | 49.4 | 59.3 | 65.4 | 75.1 | 87.0 | 67.0 | 10.3 |
|  | 2018 | 48.3 | 63.8 | 70.2 | 75.5 | 87.4 | 68.8 | 9.8 |
|  | 2019 | 45.6 | 60.9 | 69.1 | 77.0 | 87.8 | 68.4 | 10.6 |
| Precipitation  (mm) | Average | 0.0 | 1.2 | 2.9 | 5.9 | 34.2 | 4.3 | 4.7 |
|  | 2015 | 0.1 | 1.4 | 3.2 | 5.5 | 11.6 | 3.7 | 2.8 |
|  | 2016 | 0.0 | 1.8 | 3.4 | 6.6 | 20.6 | 4.7 | 4.4 |
|  | 2017 | 0.2 | 0.9 | 2.2 | 3.9 | 18.7 | 3.6 | 4.2 |
|  | 2018 | 0.0 | 1.1 | 2.8 | 6.3 | 34.2 | 4.9 | 6.4 |
|  | 2019 | 0.1 | 1.1 | 3.0 | 6.2 | 24.4 | 4.5 | 4.8 |
| Insolation  (MJ/m^2^) | Average | 4.9 | 9.8 | 13.5 | 17.0 | 24.5 | 13.6 | 4.6 |
|  | 2015 | 4.9 | 9.5 | 14.1 | 17.3 | 23.8 | 13.7 | 4.9 |
|  | 2016 | 5.0 | 8.8 | 13.2 | 17.0 | 23.6 | 13.1 | 4.5 |
|  | 2017 | 6.3 | 9.9 | 14.0 | 16.2 | 23.5 | 13.8 | 4.5 |
|  | 2018 | 6.3 | 9.9 | 12.6 | 16.6 | 24.2 | 13.5 | 4.6 |
|  | 2019 | 7.2 | 10.5 | 13.5 | 17.5 | 24.5 | 13.9 | 4.4 |
| Wind speed  (m/s) | Average | 1.3 | 1.7 | 1.9 | 2.2 | 3.3 | 2.0 | 0.4 |
|  | 2015 | 1.4 | 1.8 | 2.0 | 2.3 | 3.2 | 2.1 | 0.4 |
|  | 2016 | 1.3 | 1.8 | 2.0 | 2.2 | 3.3 | 2.0 | 0.4 |
|  | 2017 | 1.3 | 1.7 | 2.0 | 2.2 | 3.0 | 2.0 | 0.4 |
|  | 2018 | 1.3 | 1.7 | 2.0 | 2.3 | 2.9 | 2.0 | 0.4 |
|  | 2019 | 1.4 | 1.7 | 1.8 | 2.0 | 2.7 | 1.9 | 0.3 |


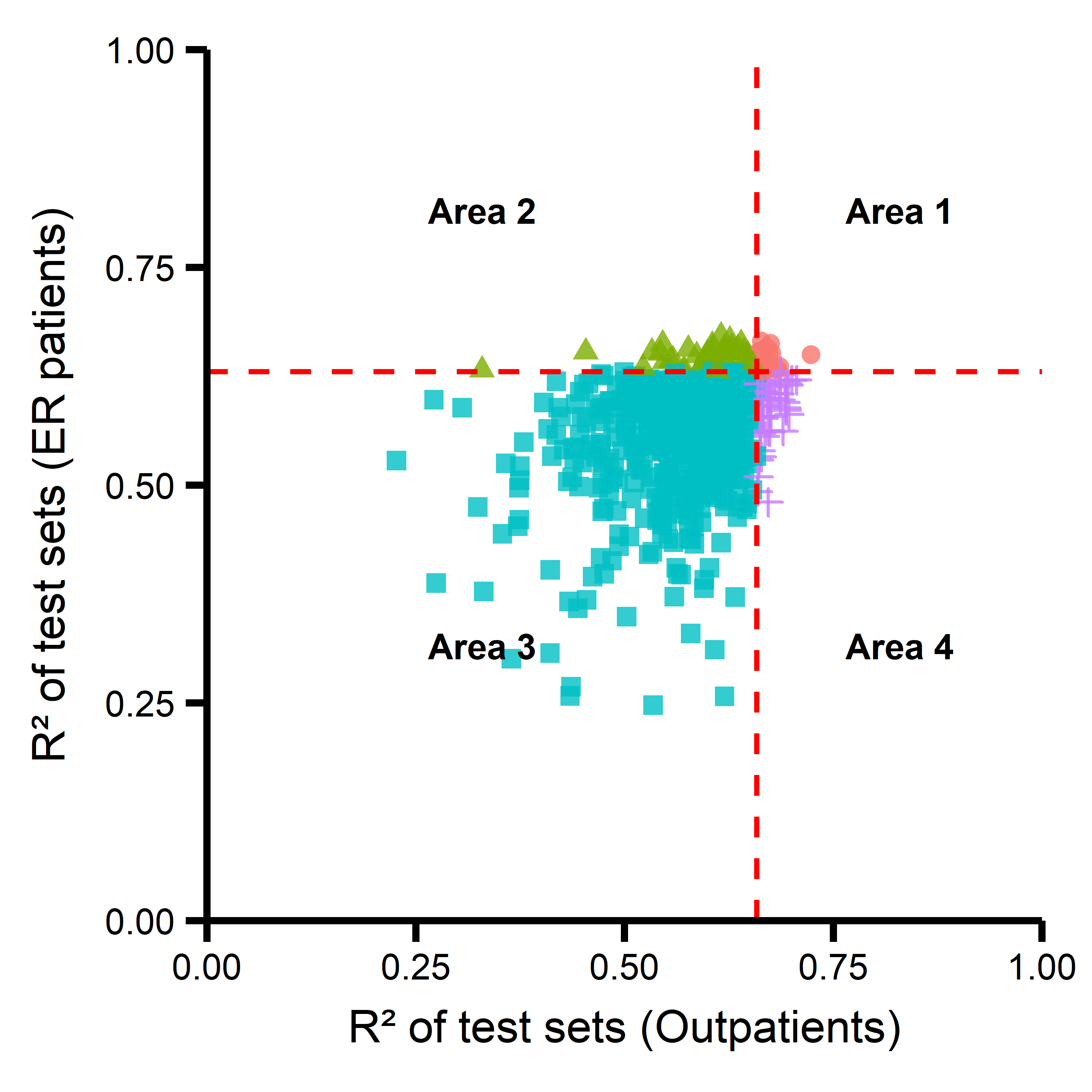


**Figure S9.** Performance (R^2^) scatter plot of modeling outpatients (x-axis) and ER patients (y-axis) for 648 models of RNN, LSTM, and GRU. The red dashed lines indicate the 90 percentile of R^2^ for outpatients and ER patients. The four areas that were divided with the red dashed lines were denoted as Areas 1 to 4.

**Table S7.** The hyperparameter values of models in Area 1 of Figure S9 and the hyperparameter values selected for the final model.

|  | **Algorithm** | **Number of units** | | | **Dropout rate** | | | **R^2^** | | |
| --- | --- | --- | --- | --- | --- | --- | --- | --- | --- | --- |
|  |  | **Layer 1** | **Layer 2** | **Layer 3** | **Input** | **Feedforward** | **Recurrent** | **Outpatient** | **ER** | **Average** |
| Models  in Area 1 | LSTM | 64 | 16 | 16 | 0 | 0.3 | 0 | 0.723 | 0.650 | 0.686 |
|  | LSTM | 64 | 16 | 8 | 0 | 0.3 | 0.1 | 0.675 | 0.663 | 0.669 |
|  | LSTM | 128 | 8 | 8 | 0.3 | 0.1 | 0.1 | 0.664 | 0.665 | 0.665 |
|  | LSTM | 128 | 8 | 8 | 0.1 | 0.1 | 0 | 0.671 | 0.658 | 0.664 |
|  | LSTM | 64 | 8 | 8 | 0 | 0.3 | 0 | 0.676 | 0.651 | 0.664 |
|  | GRU | 64 | 8 | 8 | 0 | 0.3 | 0.1 | 0.673 | 0.651 | 0.662 |
|  | LSTM | 128 | 8 | 16 | 0.1 | 0.3 | 0 | 0.686 | 0.636 | 0.661 |
|  | LSTM | 128 | 16 | 8 | 0 | 0.3 | 0.3 | 0.683 | 0.633 | 0.658 |
|  | LSTM | 128 | 16 | 16 | 0.1 | 0.3 | 0 | 0.662 | 0.653 | 0.658 |
|  | LSTM | 128 | 16 | 8 | 0.1 | 0.3 | 0.1 | 0.661 | 0.653 | 0.657 |
|  | LSTM | 64 | 16 | 8 | 0.1 | 0.1 | 0 | 0.672 | 0.639 | 0.656 |
|  | LSTM | 128 | 16 | 16 | 0.1 | 0.3 | 0.1 | 0.662 | 0.637 | 0.650 |
|  | GRU | 128 | 16 | 8 | 0.1 | 0.1 | 0.1 | 0.667 | 0.631 | 0.649 |
|  | GRU | 64 | 16 | 8 | 0.1 | 0.3 | 0.1 | 0.662 | 0.636 | 0.649 |
| Final  Model | LSTM | 64 | 16 | 16 | 0 | 0.3 | 0 | 0.723 | 0.650 | 0.686 |


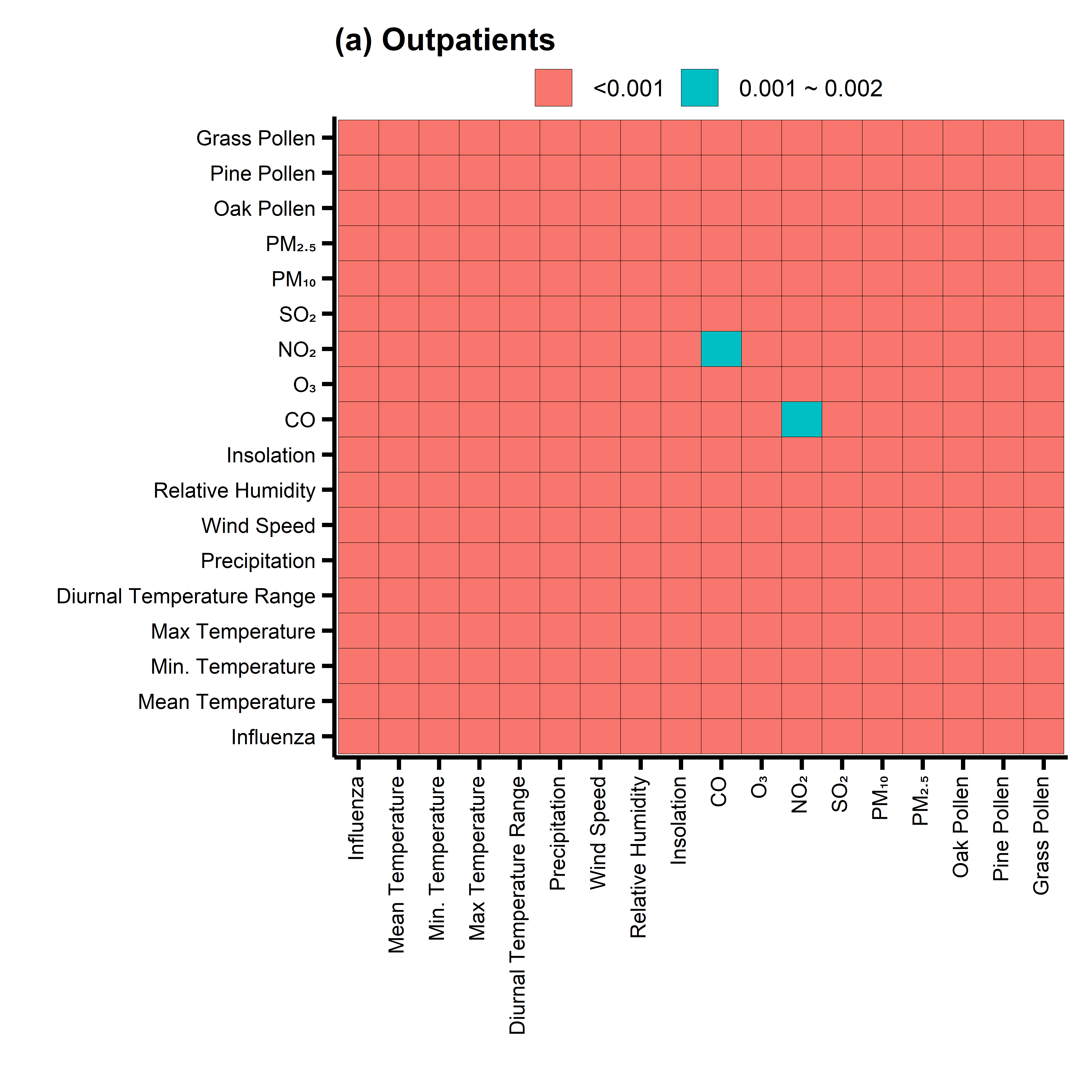

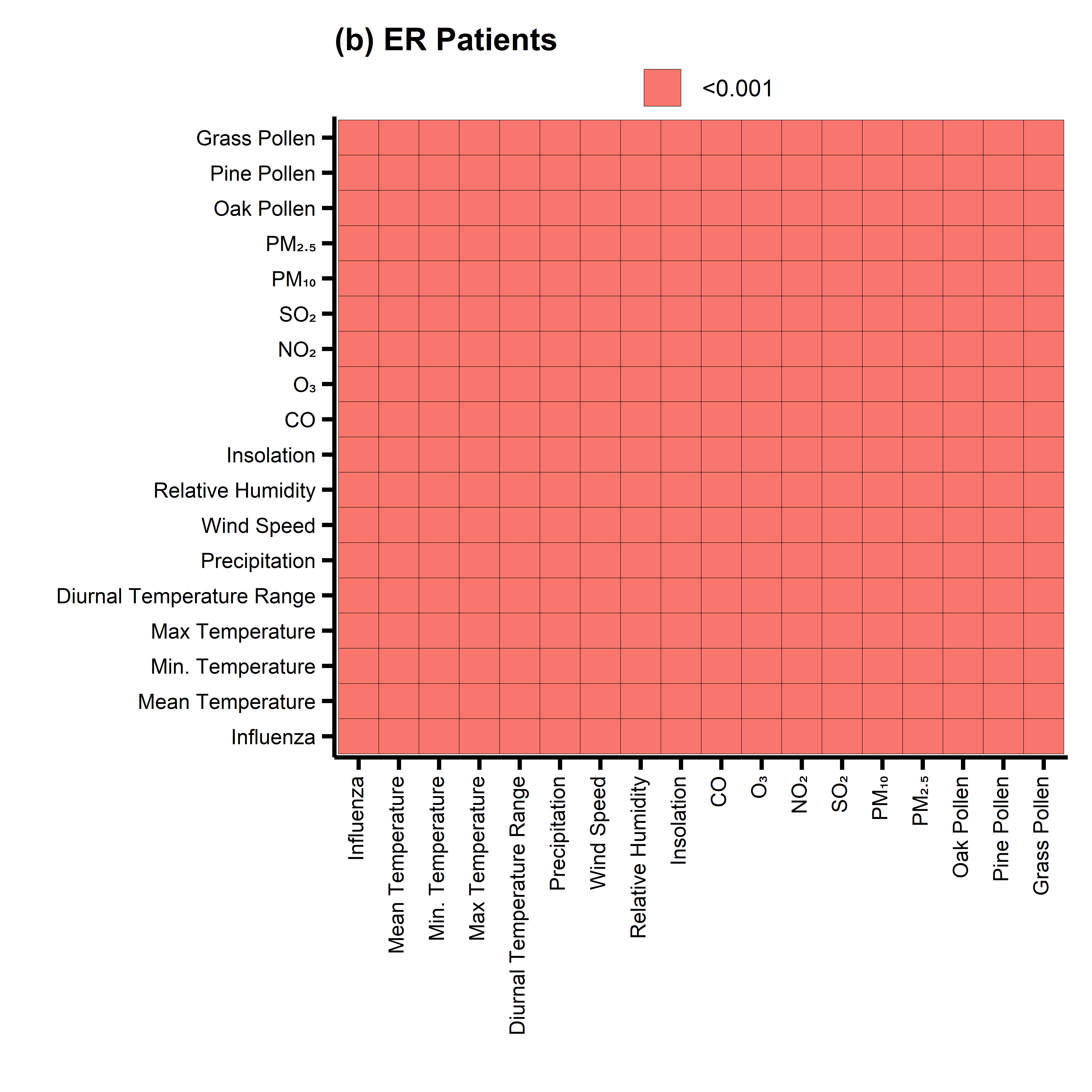


**Figure S10.** Interaction between features for (a) outpatients and (b) ER patients.
